# Free Pasture of Exotic Goats Reduces Diversity and Negatively Affects Body Condition in Scorpions (Arachnida: Scorpiones) Assemblage From Brazilian Seasonal Dry Tropical Forest

**DOI:** 10.1002/ece3.70804

**Published:** 2025-01-06

**Authors:** Thayna Rhayane Brito‐Almeida, Stênio Ítalo Araújo Foerster, José Rivaldo Lima, Meykson Alexandre da Silva, Geraldo Jorge Barbosa de Moura, André Felipe de Araujo Lira

**Affiliations:** ^1^ Programa de Pós‐Graduação Em Biodiversidade Universidade Federal Rural de Pernambuco Recife Brazil; ^2^ Departament of Zoology University of Tartu Tartu Estonia; ^3^ Programa de Pós‐Graduação Em Ciências Biológicas Universidade Federal de Pernambuco Recife Pernambuco Brazil; ^4^ Colección Nacional de Arácnidos, Departamento de Zoologia, Instituto de Biologia Universidad Nacional Autónoma de México Mexico City Mexico

**Keywords:** arachnids, community ecology, introduced herbivores, semiarid

## Abstract

Extensive grazing carried out freely by exotic goats represents an important source of anthropogenic degradation in seasonally dry tropical forests of Brazil. The presence of these herbivores may negatively impact the local fauna through the reduction of habitat complexity. In this study, we investigate the effect of goat farming in scorpion assemblage from Brazilian seasonally dry tropical forest. Scorpions were sampled in 36 areas (18 disturbed and 18 undisturbed) distributed in three sites in the seasonally dry tropical forest of Brazil. At each location, we recorded a set of local variables such as litter depth, diameter at breast height of trees, vegetation density (trees and shrubs), and detritus (stones and fallen logs). In total, 396 scorpions were collected, distributed across six species and two families. Our results showed that scorpion diversity was negatively affected, with species richness and abundance reduced in areas influenced by goats. Furthermore, in these sites, the composition of the species was also affected, with habitat‐generalist species favoring habitat‐specialist species. Finally, sites with free pasture of goats presented scorpions with reduced body condition (body mass and size) than sites without goats. Furthermore, habitat degradation caused by free pasture of goats negatively affects the assemblage of scorpions in terms of their diversity and body condition the seasonally dry tropical forest of Brazil.

## Graphical abstract

Free goat grazing impacts the Caatinga vegetation. Negatively affecting the scorpion assemblage. Furthermore, it presents context‐dependent effects on the body traits of these arachnids.

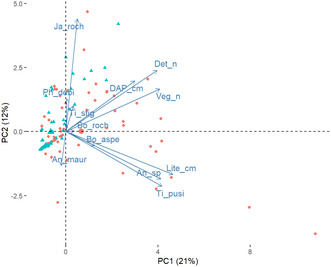

## 1 Introduction

The breeding of exotic herbivores in pasture areas is one of the oldest and most developed human activities around the world (Nori 2007). According to Steinfeld et al. (2006), land dedicated to raising these animals makes economic development viable for around 1.2 billion families. Furthermore, pastures represent more than 30% of global agricultural production (Motta‐Delgado, Martínez, and Rojas‐Vargas 2019). In Brazil, the practice is considered one of the essential sources of subsistence for countless families, especially for socioeconomically vulnerable families that live mainly in rural regions of the country (Menezes et al. 2021). For example, goats are preferred by small‐scale landless farmers because they require low initial investment and are able to digest low‐quality food as dry matter (Nair et al. 2021). In this way, goats represent one of the herbivores with greater adaptability and resistance to environmental conditions in semiarid regions (Melo 2018; Albuquerque and Melo 2018). Furthermore, much of the development of goat livestock activity in Brazil occurs in the semi‐arid region (Escareño et al. 2012). In this region, goat farming constitutes an important source of income and food for countless families (Melo 2018; Albuquerque and Melo 2018).

Despite its important socioeconomic role, goat farming is considered one of the main anthropogenic sources of degradation of native vegetation of the ecosystem (Melo 2018; Menezes et al. 2021). Following the global breeding pattern, the majority of these animals are managed through domestic breeding, exploiting native resources through free pasture (Jamelli, Bernard, and Melo 2021; Menezes et al. 2021). In this form of management, animals feed freely on native vegetation, without territorial delimitations (Melo 2018; Jamelli, Bernard, and Melo 2021). Therefore, goat grazing in native areas may cause short‐ and long‐term changes in vegetation structure, altering the natural dynamics of plant species by seedling predation, compacting the soil through constant trampling (Mor‐Mussery et al. 2021; Lins et al. 2022). Furthermore, accelerating degradation processes, slowing the regeneration process and removing the natural habitat of native species in the region (Menezes et al. 2021; Lins et al. 2022). Therefore, despite its social and economic importance, goat farming may represent an environmental risk through constant vegetation impoverishment.

The Brazilian semi‐arid region exhibits the largest portion of seasonally dry tropical forest (SDTF) biome in South America (Moro et al. 2016). This region presents distinct ecological processes and accentuated environmental characteristics, such as low annual rainfall (≤ 800 mm) and strong seasonality that directly influence the entire landscape dynamics (Medeiros et al. 2022). However, due to the intensification of human activities carried out in the region, most of the ecosystem has already been altered by chronic disturbances, due to the improper use and exploitation of its natural resources (Antongiovanni et al. 2020). According to these authors, the territory where SDTF occurs in Brazil is considered one of the country regions most vulnerable to environmental degradation.

Previous studies, carried out in Brazilian SDTF, showed that vegetation structure plays a key role in the maintenance of arthropod communities (e.g., Neves et al. 2014; Creão‐Duarte et al. 2016). Arthropods are crucial elements of the ecosystem, due to providing important ecosystem services (Ebeling et al. 2018). Among the ecosystem services of the arthropod, predation has an important influence on the health of the ecosystem (Schwab et al. 2021; Zhang et al. 2021). In semi‐arid ecosystems, scorpions stand out as arthropod predators for having high species richness and density (Polis 1990). These animals possess specific environmental requirements (Lira, DeSouza, and Albuquerque 2018) making them particularly vulnerable to changes in their habitat. For example, in the Brazilian SDTF, areas with higher vegetation complexity harbor a greater number of species of these arachnids than areas with low complexity (Foerster, Lira, and Almeida 2020; Lira, Araujo, et al. 2021). According to these authors, the reduction in vegetation complexity caused by human actions such as opening of roads and deforestation favors generalist species to the detriment of habitat‐specialist species. Furthermore, habitat change negatively influences the body condition of scorpions, where in degraded areas they present smaller individuals when compared to individuals from preserved areas (Lira et al. 2020).

Therefore, despite being an important economic activity for low‐income populations, goat farming negatively impacts native biodiversity. While the effects of this practice on vegetation are already well documented, what are the specific effects of free pasture of goats on invertebrates that are closely influenced by vegetation structure? In this study, we evaluated the impacts of free pasture of goats on scorpion assemblages from Brazilian SDTF. Specifically, we analyzed the effects of free pasture of goats on scorpion diversity (species richness, abundance, and composition) and body condition (body size and mass). Testing the following hypotheses: (i) areas with the free pasture of goats will present a reduction in the richness and abundance of scorpions, (ii) the presence of goats will favor the presence of generalist species to the detriment of habitat‐specialist species, and (iii) scorpions for the areas with free pasture of goats, they will present reduced body condition (size and mass).

## 2 Materials and Methods

### 2.1 Sampling Procedures

Fieldwork was conducted between May and August 2022 in the three municipalities in Pernambuco state (Caetés, Cumaru and Limoeiro) with similar landscape inserted in the Brazilian SDTF. Annual rainfall at the sampling sites ranges from 400 to 652 mm, and the temperature ranges from 21.5°C to 24.8°C (Climate Data 2024). The studied areas were classified as hyperxeroophic Caatinga characterized by the shrubby‐arboreal vegetation. Each site was sampled during the rainy season (rainfall ranges from 43 to 48 mm) due to the increased foraging activity of scorpions (Lira, DeSouza, and Albuquerque 2018).

In each sampling municipality, areas of native vegetation were selected a distance from each other of at least 500 m, six of which were used for free pasture of goats (hereafter, disturbed) and six without the presence of these animals (hereafter, undisturbed). The undisturbed areas had no known disturbance factors (fire, firewood, or pole harvest), while in the disturbed areas the only known factor was the presence of goats. In each area (disturbed and undisturbed), four transects (30 m long) were established as sampling units. Transects were 20 m apart from each other. Scorpions located up to 5 m away from both sides of each transect were collected, resulting in 14,400 m^2^ of sampled area per site. The sampling was carried out at night, between 19:00 and 21:00 h, and scorpions were captured with the aid of tweezers. Each transect was conducted during 1 h by a pair of collectors equipped with ultraviolet flashlights. All scorpions recorded in the transects were collected, individually labeled, and stored in 70% ethanol. Specimens were identified according to Lourenço (2002) and Esposito et al. (2017). For each adult individual, the following parameters were measured as body condition: fresh body mass obtained using a precision digital scale (0.001 g), the length of prosoma, metasoma V segment, movable finger, and width and height of the pedipalp chelae through a digital caliper (0.01 mm). The voucher specimens were deposited in the Arachnological Collection of the Universidade Federal de Pernambuco, Brazil.

### 2.2 Environmental Characterization

We adapted the procedures used by Lira, Araujo, et al. (2021) to characterize the Caatinga areas; therefore, four local variables were measured inside each transect area. (i) Vegetation: all live vegetation > 1.5 m (trees) and 1 m (shrub) above ground that touched the transect tape was recorded; (ii) diameter at breast height (DBH): for each tree (with more than 1.5 m above ground) counted, the DBH was measured with a tape; (iii) detritus: all stones with a diameter > 5 cm and all logs with a length > 10 cm were counted; (iv) depth of the leaf litter: the corners of four quadrats (25 × 25 cm) were measured.

### 2.3 Data Analyses

Initially to visualize the environmental structure of disturbed and undisturbed areas, a principal component analysis (PCA) was carried out. The effects of local variables on the species richness and abundance of scorpions in disturbed and undisturbed areas were analyzed using generalized linear models (GLM) applying Poisson (species richness) and negative binomial (abundance) distributions. The variable “goat” was added as a predictor variable and before analysis; the other local variables were log‐transformed. The models were reduced based on the Akaike information criterion (AIC), where the predictor variables excluded from models with lower AIC were considered no significant. The normality of residuals was visually assessed using Q–Q plots, and the presence of outliers was tested using Cook's distance. GLMs were performed in the R software using the MASS package (R Development Core Team 2023).

The variation in species composition between disturbed and undisturbed areas was analyzed using the percentage dissimilarity index (Legendre and De Cáceres 2013) using a multivariate analysis of permutational variance (PERMANOVA) through 1000 permutations (McArdle and Anderson 2001). Then the effect of local variables on species composition for each area (disturbed and undisturbed) was carried out using a distance‐based redundancy analysis (dbRDA) (McArdle and Anderson 2001). The significance of the relationships was analyzed using 1000 permutations. These analyzes were performed in R software using the vegan package (Oksanen et al. 2019).

Finally, the effect of environmental variables and free pasture of goat on scorpion body condition was analyzed using linear models (LM). A priori, body condition metrics (fresh body mass, length of prosoma, metasoma V segment, movable finger, and width and height of the pedipalp chelae) were log‐transformed and then summarized on a single axis of a principal component analysis (PCA). Through this approach, we assume that axis 1 of the PCA captures the overall size of specimens (Foerster et al. 2024). The values of axis 1 of the PCA were used as the response variable and the local variables were used as predictor in our LMs. These analyzes were performed using R software (R Development Core Team 2023).

## 3 Results

**FIGURE 1:**
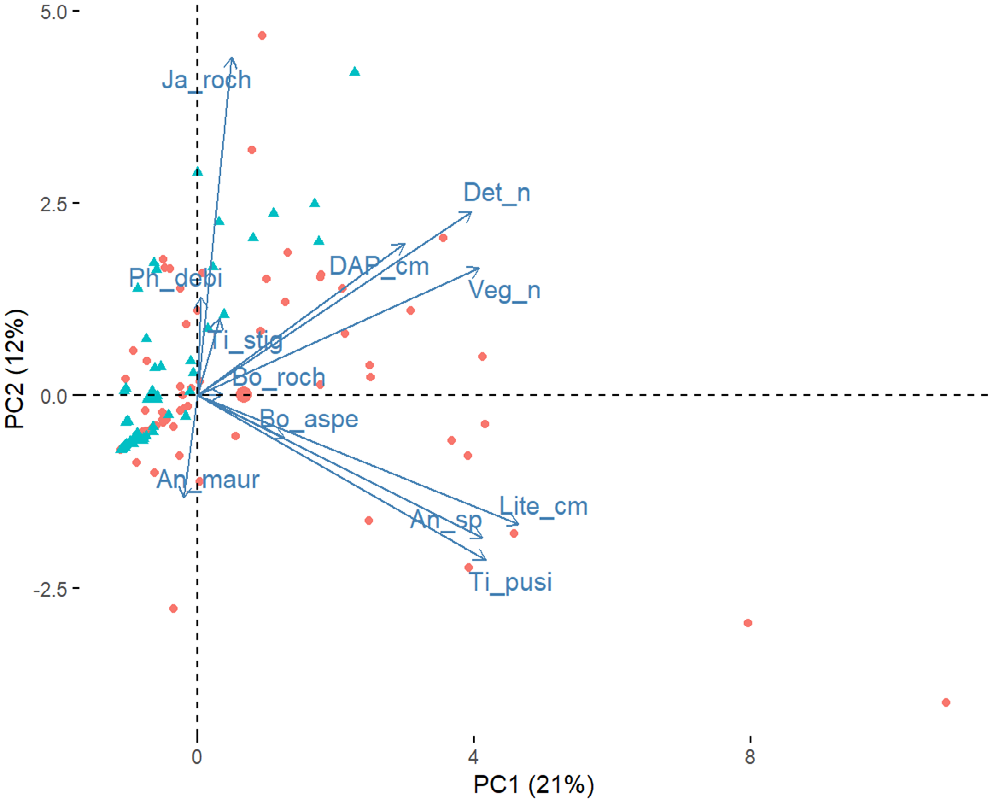
Variation in the structure of the environment and composition of scorpion species according to goat farming in the Brazilian seasonal dry tropical forest. Red points = undisturbed sites, green triangles = disturbed sites. An_maur, *A. mauryi*; An_sp, *Ananteris* sp.; Bo_aspe, *B. asper* ; Bo_roch, *B. rochai*; DAP_cm, diameter at breast height (in cm); Det_*n*, number of stones and fallen logs; Ja_roch, *Jaguajir rochae*; Lite_cm, leaf litter depth (in cm); Ph_debi, *P. debilis* ; Ti_pusi, *T. pusillus* ; Ti_stig, *T. stigmurus*; Veg_*n*, number of trees and shrubs.

The first two axes of the PCA retained 37% of the variation, showing that the disturbed and undisturbed areas shows differences in their local variables (Figure 1). Disturbed areas showed lower values of local variables compared to undisturbed areas (Table 1). In total, 396 scorpions were collected, distributed among six species of Buthidae and two species of Bothriuridae (Table 2). *Jaguajir rochae* (Borelli 1910) (*n* = 140 individuals), *Bothriurus rochai* Mello‐Leitão 1932 (*n* = 95), *Tityus pusillus* Pocock 1983 (*n* = 65) and *Ananteris mauryi* Lourenço 1982 (*n* = 57) represented 89% of the total sampled. The other species, *Ananteris* sp. (*n* = 19), *B. asper* Pocock 1893 (*n* = 9), *Physoctonus debilis* (C.L. Koch 1840) (*n* = 7), and *T. stigmurus* (Thorell 1876) (*n* = 4) corresponded to 11% of the sampling.

**TABLE 1:** Environmental structure in Brazilian seasonal dry tropical forest areas with the presence and absence of goats.

Local variables	Vegetation area	
	Goat absence	Goat presence
Leaf litter depth (cm)	0.34 ± 0.77	0
DBH (cm)	7.09 ± 11.02	3.19 ± 9.01
Vegetation (*n*)	24.58 ± 16.93	5.51 ± 13.23
Detritus (*n*)	25.52 ± 35.20	14.16 ± 22.40

Abbreviation: DBH, diameter at breast height.

**TABLE 2:** Diversity of scorpions in Brazilian seasonal dry tropical forest areas with the presence and absence of goats.

Scorpion species	Pasture	
	Goat absence	Goat presence
Buthidae		
*Ananteris* sp.	19	0
*Ananteris mauryi* Lourenço 1982	57	0
*Jaguajir rochae* (Borelli 1910)	73	67
*Physoctonus debilis* (C.L. Koch 1840)	1	6
*Tityus pusillus* Pocock 1893	65	0
*Tityus stigmurus* (Thorell 1876)	3	1
Bothriuridae		
*Bothriurus asper* Pocock 1893	8	1
*Bothriurus rochai* Mello‐Leitão 1932	29	66

The number of species ranged from 3 to 7 in scorpion assemblages in undisturbed areas and from 0 to 4 species in disturbed areas. The species richness of these arachnids was sensitive to variation in the structure of the environment. In the reduced model, the local variables DBH and detritus were retained. These variables positively affected the richness of scorpion species (DBH: Estimate = 0.1927, *p* = 0.0066; detritus: Estimate = 0.2668, *p* = 0.0002). In terms of abundance, the variation was 63–126 and 0–100 individuals for areas without and disturbed, respectively. The abundance of scorpions was also sensitive to variations in the structure of the environment. In the reduced model, the local variables leaf litter depth, vegetation, and detritus were retained. However, only the detritus was significantly and positively affected the abundance of scorpions (Estimate = 0.3519, *p* = 0.0005).

The composition of scorpions differed between areas with the presence and absence of goats (PERMANOVA: *F*_1_,_74_ = 2.0824, *p* = 0.0179). *Tityus pusillus* and *Ananteris* spp. were absent in disturbed areas presence (Table 2). Furthermore, the structure of the habitat influenced the composition of scorpions in disturbed and undisturbed areas differently. In areas with the presence of goats, the variation in composition was explained by detritus (*F* = 2.7321, *p* = 0.0059, *R* ^2^ = 0.0743) while in the absence of goats, the variation in the composition of scorpion species was explained by the depth of the leaf litter (*F* = 4.1631, *p* = 0.0009, *R* ^2^ = 0.1011).

Due to the number of individuals collected in disturbed and undisturbed, body condition analyze were carried out for the species *J. rochae* and *B. rochai*. The axis 1 of the PCA captured 52.3% and 48.6% of the size variation in the body condition of *J. rochae* and *B. rochai*, respectively. For both species, body condition were negatively positioned with PC1 (Table 3) and responsive to local variables (*J. rochae*: *F*_1_,_90_ = 7.5140, *p* = 0.0073; *B. rochai*: *F*_3_,_52_ = 4.9710, *p* = 0.0041). Detritus was negatively related to PC1 for *J. rochae* (Estimate = −0.4526, *p* = 0.0074), indicating that areas with a greater amount of detritus support larger individuals. For *B. rochai*, negative relationships were found between PC1 and DBH (Estimate = −0.4844, *p* = 0.0182) and the presence of goats (Estimate = −1.5558, *p* = 0.0174) and a positive relationship with the amount of detritus (Estimate = 0.4534, *p* = 0.0281).

**TABLE 3:** Principal components analysis (PCA) of body condition of the scorpion species *Jaguajir rochae* (Borelli 1910) and *Bothriurus rochai* Mello‐Leitão 1932.

Body traits	*Jaguajir rochae*		*Bothriurus rochai*	
	PC1	PC2	PC1	PC2
Fresh mass	−0.401	−0.274	−0.398	0.004
Prosoma length	−0.378	−0.417	−0.085	−0.901
Metasoma V segment length	−0.468	−0.023	−0.337	0.379
Pedipalp chela length	−0.169	−0.403	−0.393	−0.097
Pedipalp chela height	−0.414	0.524	−0.460	−0.151
Pedipalp chela width	−0.425	0.495	−0.407	0.113
Movable finger length	−0.312	−0.261	−0.435	0.017

## 4 Discussion

In the present work, we evaluated the effect of the free pasture of goats in areas of native vegetation on the scorpion assemblage of Brazilian SDTF. Our results show differences in the structure of the environment (37% variation) between disturbed and undisturbed areas. Undisturbed environments have a higher number of detritus (rocks and fallen logs) and vegetation. Trampling by exotic herbivores in dry regions causes surface breaking and shaking (Warren et al. 1986). According to these authors, trampling causes soil debris to break down, mainly rocks and boulders, resulting in an increase in surface irregularity and making it difficult for water to infiltrate the soil. Furthermore, goat trampling may negatively affect plant establishment (Kidron 2016). In the Brazilian SDTF, goats represent a generalist consumer of native vegetation, being responsible for the removal of numerous plant species (Pfister, Malechek, and Balph 1988; Menezes et al. 2021). Given their high voracity, goats are capable of significantly reducing herb biomass and seedlings survival in regions of the Brazilian semi‐arid region (Menezes et al. 2021; Lins et al. 2022). Therefore, the presence of grazing goats in the Brazilian SDTF areas is capable of modifying the structure of the vegetation, reducing the complexity of the environment.

Our results indicated that the environmental changes caused by the free pasture of goats influenced the species richness and abundance of scorpions. According to Foerster, Lira, and Almeida (2020), increases in environmental heterogeneity increase the abundance and richness of scorpion species supported by the site. The density of detritus (stones and fallen logs) was positively related to the species richness and the abundance of scorpions. Considering that the Brazilian SDTF is an open forest formation with arboreal‐shrubby vegetation, the detritus may act in two ways to maintain the scorpion assemblage. (i) Provide food resources for animals, as in dry forests there is a positive relationship between environmental complexity and the number of invertebrates (Neves et al. 2014; Sousa‐Souto et al. 2014; Leal et al. 2016). Previous studies reported the use of 12 groups of invertebrates as prey by Brazilian SDTF scorpions, including other scorpions and small vertebrates (Dionisio‐da‐Silva et al. 2024); (ii) may prevent encounters between individuals and thus reduce intraguild predation and cannibalism. Habitat structure plays an important role in antagonistic relationships between predators (e.g., Bellone, Klapwijk, and Björkman 2017; Moreira et al. 2022). In an experimental laboratory study, it was shown that antagonistic intra‐ and interspecific interactions of scorpions were reduced with increasing arena complexity (Moreira et al. 2022). Therefore, free pasture of goats in areas of native vegetation is capable of indirectly influencing the diversity of the scorpion assemblage in the Brazilian SDTF.

In addition to the reduction in species richness and abundance, areas impacted by the presence of goats presented a composition of scorpion species different from those undisturbed areas. For example, habitat‐specialist species such as *T. pusillus* and *Ananteris* spp. (Lira, DeSouza, and Albuquerque 2018) were found only in undisturbed areas with availability of leaf litter. These animals have a small body size and, like other arthropods, are easily susceptible to desiccation (Lira, Foerster, et al. 2021; Nervo et al. 2021). For these scorpions, leaf litter, by maintaining humidity, plays a crucial role in their survival, especially in a region with high temperature and evapotranspiration such as the Brazilian SDTF (Janzen and Schoener 1968; Barrientos 2012; Bujan, Yanoviak, and Kaspari 2016). For disturbed areas, our results showed that the amount of detritus influenced the composition of species. In open forest areas, the amount of detritus may represent the availability of shelter. Scorpions have a size‐structured assemblage with larger individuals acting as predators of smaller ones (Moreira et al. 2022). Therefore, the presence of larger amounts of detritus in a given location may favor the permanence of small species such as *B. asper* . On the contrary, the scorpions *J. rochae* and *B. rochai* were the most abundant species in the samples, occurring in disturbed and undisturbed areas. These two species correspond to medium‐large animals capable of colonizing a large part of the available microhabitats (Lira, DeSouza, and Albuquerque 2018; Lira, Foerster, et al. 2021) and are therefore capable of occurring even in degraded areas (Foerster, Lira, and Almeida 2020).

Despite the occurrence of *J. rochae* and *B. rochai* in disturbed areas, body condition showed a species‐specific response to the presence of herbivores. Our results showed a variation of 52.3% and 48.6% in the body condition of *J. rochae* and *B. rochai*, respectively. Individuals of *J. rochae* demonstrated a decrease in their body condition related to the reduction in detritus. Habitat modification negatively affects the distribution of potential prey, thus predatory arthropods end up being directly harmed by the decrease in resource supply (Chen and Wise 1999). Arthropods exposed to food shortages during the beginning of their development may show body reduction as adults (Filgueiras et al. 2015). Similar results were described for spiders and scorpions exposed to an environment with low prey availability, showing a decrease in their body size (Lichtenstein et al. 2016; Lira et al. 2020). According to Kotiaho et al. (1999) individuals that have a decrease in body condition have a short life cycle, either because they have low resources to escape predators or difficulty in capturing food. Therefore, the environmental changes caused by the free pasture of goats of native vegetation may negatively impact the population of *J. rochae*.

For *B. rochai* populations, individuals with higher body condition were found in sites with trees with higher DBH values and disturbed areas. However, lower values of body condition were related to an increase in the amount of detritus. According to Lira, Araujo, et al. (2021) individuals of *B. rochai* dig their burrows close to vegetation in protected locations, which may avoid encounters with potential predators. Therefore, trees with higher DBH may favor the presence of larger individuals of *B. rochai* in the Brazilian SDTF areas. In addition, the rate of predation suffered by this species may be behind the relationship between the presence of goats and the amount of detritus with body condition. Polis, Sissom, and McCormick (1981) suggested that for arid regions, the main predators of scorpions are other larger scorpions. In the Brazilian SDTF region, individuals of *B. rochai* are frequently preyed upon by individuals of *J. rochae* (Lira person. obs.). Therefore, considering that areas with few detritus and the presence of goats negatively impact the *J. rochae* population, these impacts may be beneficial for *B. rochai* by decreasing the efficiency of an intraguild predator, thus allowing greater values of body condition.

## 5 Conclusions

In summary, our results showed that areas of native vegetation where free pasture of goats occurs have lower local complexity compared to undisturbed areas. This change in local complexity negatively influences the scorpion assemblage, resulting in a decrease in species richness and abundance. Additionally, we observed a replacement of habitat‐specialist species by habitat‐generalist species. These findings have significant implications for biodiversity conservation in semi‐arid regions. The reduction in scorpion richness and abundance may impact ecological balance, as these organisms play important roles in ecosystems, such as controlling invertebrate populations. Beyond diversity, the body condition of scorpions was also influenced by free pasture of goats, highlighting a species‐specific response. This suggests that different species may have varying tolerances to human‐induced changes, which is crucial for understanding community dynamics. Considering that goat farming constitutes an important source of income for many families in semi‐arid regions, we advocate for the implementation of new management methods aimed at mitigating the negative effects of livestock farming on native fauna. Strategies such as rotational grazing could be essential for balancing economic needs with environmental conservation.

## Author Contributions

**Thayna Rhayane Brito‐Almeida:** conceptualization (equal), data curation (equal), methodology (equal), writing – original draft (equal). **Stênio Ítalo Araújo Foerster:** data curation (equal), formal analysis (equal), validation (equal), visualization (equal), writing – review and editing (equal). **José Rivaldo Lima:** data curation (equal), investigation (equal), resources (equal), writing – review and editing (equal). **Meykson Alexandre da Silva:** data curation (equal), investigation (equal), resources (equal), writing – review and editing (equal). **Geraldo Jorge Barbosa de Moura:** conceptualization (equal), funding acquisition (equal), methodology (equal), writing – review and editing (equal). **André Felipe de Araujo Lira:** conceptualization (equal), investigation (equal), methodology (equal), project administration (equal), validation (equal), visualization (equal), writing – review and editing (equal).

## Conflicts of Interest

The authors declare no conflicts of interest.

## Supporting information


Data S1.

## Data Availability

The dataset used in this study is available in Supporting Information.
